# Local Recurrence Risk Score to Predict Relapse after Stereotactic Body Radiation Therapy for Lung Tumors

**DOI:** 10.3390/jcm11216445

**Published:** 2022-10-31

**Authors:** Isabelle Pougnet, Paul Habert, Sarkis Delcourt, Mohamed Boucekine, Stephanie Wong, Audrey Zacchariotto, Xavier Muracciole, Jean-Yves Gaubert, Laetitia Padovani

**Affiliations:** 1 Oncology Radiotherapy Department, CRCM Inserm, UMR1068, CNRS UMR7258, AMU UM105, Genome Instability and Carcinogenesis, Assistance Publique des Hôpitaux de Marseille, Aix Marseille University, 13284 Marseille, France; 2 Imaging Department, Hopital Nord, APHM, Aix Marseille University, 13284 Marseille, France; 3 Aix Marseille University, LIIE, 13284 Marseille, France; 4 Aix Marseille University, CERIMED, 13284 Marseille, France; 5 Service de Médecine Nucléaire, Hôpital La Timone, Rue Saint Pierre, Aix Marseille University, 13005 Marseille, France; 6 EA 3279: CEReSS—Health Service Research and Quality of Life Center, School of Medicine—La Timone Medical Campus, Aix Marseille University, 13005 Marseille, France; 7 Support Unit for Clinical Research and Economic Evaluation, Department of Clinical Research and Innovation, Assistance Publique—Hôpitaux de Marseille, 13385 Marseille, France; 8 Service de Radiologie, Hôpital La Timone, 264 Rue Saint Pierre, 13005 Marseille, France

**Keywords:** stereotactic radiations, follow up studies, non-small-cell lung carcinomas, CT scanner, X-ray

## Abstract

Background: After stereotactic body radiation therapy (SBRT) for lung tumors, follow-up CT scans remain a pitfall. The early detection of local relapse is essential to propose a new treatment. We aim to create a local recurrence predictive score using pre- and post-therapeutic imaging criteria and test it on a validation cohort. Methods: Between February 2011 and July 2016, lung tumors treated by SBRT with available pretreatment fluorine-18-fluorodeoxyglucose positron emission tomography (FDG-PET) and follow-up CT scans were retrospectively analyzed. The risk factors associated with relapse were identified by univariate logistic regression on a train cohort. The score was created using these factors, merging clinical and imaging criteria associated with local relapse, and then tested on an independent validation cohort. Overall and local relapse-free survival at 1 and 3 years were recorded. Results: Twenty-eight patients were included in the train cohort and ten in the derivation cohort (male 74%, median age 70 ± 12 years). Five variables significantly associated with local recurrence (female gender; sequential enlargement; craniocaudal growing; bulging margins; standardized uptake value (SUV_max_ > 5.5)) were combined to create the score on five points. With the threshold >2.5/5, the sensitivity and specificity of the score on the validation cohort were 100% and 88%, respectively. Overall survival and local relapse-free survival at 1 and 3 years were 89% and 42%, and 89% and 63%, respectively. Conclusion: The local recurrence risk score created has high sensitivity (100%) and specificity (88%), upon independent validation cohort, to detect local relapse. This score is easy to use in daily clinical practice.

## 1. Introduction

Lung stereotactic body radiation therapy (SBRT) is an innovative technique currently only indicated for selected patients. This technique allows the delivery of high doses of radiation to a small target volume, in a small number of fractions, with millimeter accuracy. The risk to adjacent organs is reduced thanks to a strong dose gradient.

The American Society for Radiation Oncology guidelines have recommendations about non-small-cell lung cancer (NSCLC) stage 1A, where the first treatment for medically operable patients is surgical removal with mediastinal lymph node dissection or sampling [1]. Lung SBRT should be used as an alternative for patients who are unfit for surgical resection. Unfortunately, randomized trials comparing surgery and SBRT closed early because of poor accrual [2]. The role of SBRT in the treatment of surgical candidates is under investigation and the proportion of borderline or potentially operable patients who receive SBRT for early-stage lung cancer is expected to increase [3,4]. Thanks to a biological equivalent dose exceeding 100 Gy, this technique has demonstrated a 3-year local control rate for early-stage, inoperable NSCLC of more than 90%. Local control rates reached 86% at 5 years and overall survival of 47% at 5 years [5]. SBRT for lung oligometastatic disease is also safe and effective, with local control rates of about 80% [6].

Although the local recurrence rate is very low, the early detection of local relapse is crucial to propose a new treatment. If feasible, these include radiofrequency, surgical resection, targeted agents for patients harboring oncogenic driver mutations, or, in selected patients, repeated irradiation. However, post-therapeutic chest computed tomography (CT) changes could be confusing due to the presence of benign radiation-induced lung injury, which can mimic local recurrence [7]. Benign and acute post-stereotactic lung lesions appear in 60% of cases within 6 months, and in 90% of cases after 6 months [8]. The Response Evaluation Criteria in Solid Tumors [9] score usually used in oncology does not seem suitable. In this context, it seems important to take advantage of additional elements, which help clinicians in cases of suspected local recurrence after lung SBRT. Metabolic data appear to be promising, but their role is currently unclear. We thought fluorine-18-fluorodeoxyglucose positron emission tomography (FDG-PET) performed prior to SBRT, combined with a post-treatment follow-up CT scan, could help to find local tumor recurrence criteria.

The aim of the study was to find pre- and post-therapeutic clinical and imaging risk factors for local recurrence and then create a predictive score to predict local recurrence.

## 2. Materials and Methods

### 2.1. Patients

We retrospectively reviewed a single-institution clinical database of patients treated by SBRT for early-stage primary lung cancer or lung oligometastases of any primary localization. In total, 102 consecutive patients were treated in our university hospital between February 2011 and July 2016. Having a PET-CT scan within 3.5 months prior to the start of stereotactic radiation was required for inclusion. Histological evidence was not required if the medical risk of biopsy was considered high. If no histological evidence was reasonably possible, radiological, metabolic, and clinical parameters were necessary for decision making. All radiation therapy decisions had to be validated by a multidisciplinary team including thoracic surgeons, medical oncologists, radiation oncologists, pathologists, and radiologists. Patients were excluded from analysis if their regular monitoring imaging after treatment was not accessible for review. Toxicity data were graded according the Common Terminology Criteria for Adverse Events V4.03 [10]. All patients approved the study and signed an informed consent form.

### 2.2. SBRT

SBRT was performed with the continuous helical delivery of 6MV photons using the Accuray^®^ TomoTherapy^®^ system. Abdominal compression was applied to reduce tumor excursion during the respiratory cycle. We realized three daily consecutive scans with abdominal compression. The internal treatment volume was defined as the union of the three different gross tumor volumes. The planning target volume was defined by internal tumor volume plus 0.5 cm. Image-guided radiotherapy by megavoltage computed tomography was performed daily and medically approved online. The dose prescription was adapted to tumor localization: central or peripheral. During the period of inclusion, the prescribed radiation doses were not homogeneous. The biologic effective dose (BED) was calculated using the following formula:

$$BED_{10}=n\cdot d\left(1+\frac{d}{\alpha /\beta }\right)$$

where *n* is the number of fractions, *d* is the dose of one fraction, and the alpha/beta value is 10 Gy.

### 2.3. FDG-PET Procedure and Parameters

The FDG-PET scan was reviewed to determine the metabolic data of all lesions and used to decide on the appropriate treatment. The blood glucose level was monitored prior to injection of the contrast agent. All metabolic data were retrospectively collected by a single nuclear medicine physician (SD). The regions of interest (ROIs) were manually drawn on the FDG-PET acquisitions merged with the CT scan in axial view on a dedicated software (AW server 4.6, GE Healthcare, Milwaukee, WI, USA). The physician visually checked that the most fixing voxel was indeed present in the ROI. If not, the ROI was re-adapted in the three-dimension plans. This technique improves reproducibility and dramatically reduces error. The standardized uptake value (SUV) is defined by:

$$SUV=\frac{r}{a^{\prime }/\;W}$$

where *r* is the radioactivity concentration (kBq/mL) measured within a ROI, *a*′ is the decay-corrected amount of injected radiolabeled FDG (kBq), and *W* is the weight of the patient (g).

SUV_max_ is the highest value of SUV in a pixel of the defined ROI. SUV_mean_ is the mean value of SUV in the defined ROI. SUV_peak_ is defined as the mean value of pixels in a small sub-ROI of 10 mm in diameter, within the originally circled ROI automatically centered on the SUV_max_. We then investigated the relative metabolic tumor volume (MTV) threshold. MTV 42% is defined as the volume within the ROI with SUV >42% of SUV_max_. The lesion glycolysis peak was calculated by multiplying MTV and SUV_peak_.

### 2.4. CT Scan Monitoring

A review of the literature identified high-risk CT imaging features that could help in distinguishing local recurrence from post-treatment fibrosis. The radiologic criteria are as follows, including seven high-risk features [11,12,13,14,15]: enlarging opacity; sequential enlarging opacity (defined as three increases in lesion volume at three-month interval); enlarging opacity after 12 months; craniocaudal direction growth; bulging margin; linear disappearance; loss of air bronchogram; ipsilateral pleural effusion; and lymph node enlargement.

All of the follow-up CT scans of each patient were retrospectively and anonymously reviewed by one radiologist with 8 years of experience in thoracic imaging (PH), blinded to the local recurrence status.

### 2.5. Local Relapse Definition

All follow-up scans were reviewed by a radiation oncologist specialized in pulmonary radiation therapy. A reduction or no change in the tumor volume after SBRT classified patients as non-recurrent. Local recurrence was defined as a biopsy-proven relapse or radiological certainty of relapse. The latter was more difficult to assess. Due to the retrospective design, the patient’s disease history was helpful in all cases of non-biopsy-proven relapse: if there was a growing lesion in the planning target volume (PTV), a PET-CT was performed and its positivity led to classify the patient as being suspected of local recurrence. Among these, if this suspicion of local recurrence led to any new cancer treatment decided by the multidisciplinary tumor board, or if distant metastasis appeared in the follow-up, the patient changed classification to certainty of local recurrence. Otherwise, the medical and radiological history was reviewed by a chest radiologist with 8 years of experience (PH). This radiologist classified the cancer as a recurrence or not, depending on whether there was regional nodular contrast uptake in the radiation scar. Having all of the successive thoracic CT scans of each patient helped with classification. However, some patients were classified as “unknown” if the local appearance did not distinguish a local relapse from a radiation-induced lung injury.

### 2.6. Independent Validation Cohort

To evaluate the performance of the score, we then tested it on a retrospective patient cohort from consecutive patients of the same institution treated by SBRT after July 2016 and following the same inclusion criteria as the train cohort. This included ten patients with two proven local relapses (two biopsies). Pretreatment PET scans and post-treatment thoracic CT scans of this cohort were submitted to one nuclear medicine physician (SD) and one radiologist (PH), blinded to the local recurrence status.

## 3. Statistical Analysis

Categorical variables are presented as numbers and percentages, and numerical variables are presented as median ± interquartile range (IQR). The significance of baseline differences was determined by chi-square test, Fisher’s exact test, or the unpaired t-test, as appropriate. The most relevant risk factors associated with relapse were identified by univariate logistic regression analysis. Because there were multiple dates involved in the score, this did not allow us to consider a single time-to-event relapse; consequently, a Cox regression was not used. A multivariate regression was not suitable because of the small sample and few events. The odds ratios (OR) and 95% confidence intervals (CI) are shown. Patient characteristics, pre-stereotactic lung radiation therapy metabolic data, and post-treatment scan data were included in the univariate analyses. Significant variables in the univariate analysis were used to compute an aggregate score. One point was assigned to each variable if it was present, with the scores ranging from zero to five points. A receiver operating characteristics (ROC) curve was generated and Youden’s index was used to determine the threshold for which (sensitivity + specificity) was maximal. This threshold was then tested on the validation cohort. Local recurrence-free survival curves were drawn according to this threshold and compared using the log-rank test. To assess the importance of different items in the score, we used the variable importance measure derived from the Random Forest algorithm [16], after having trained the model on the train cohort. Statistical significance was set at *p* < 0.05.

## 4. Results

### 4.1. Patients and SBRT Characteristics

**Figure 1:**
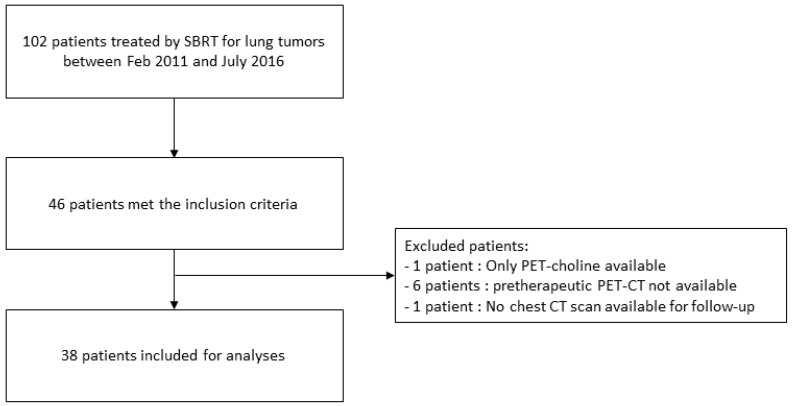
Flow chart of the study.

Between February 2011 and July 2016, 102 patients were treated by lung SBRT in our university institution. Forty-six of them met the inclusion criteria. Thirty-eight patients were analyzed in our retrospective cohort (Figure 1). The patient characteristics are shown in Table 1. Among the 38 analyzed patients, survival and progression-free data could be analyzed for 34. For the four remaining patients, the local recurrence state was unclear because of doubt between local relapse and radiation-induced lung injury. The median time between the PET scan and radiation therapy was 60 days. The total dose prescription ranged from 16 Gy to 60 Gy, in three to nine fractions. The median BED_10_ for an alpha/beta of 10 was 78 Gy [48–132]. The median equivalent dose in 2 Gy fractions (EQD2) was 65 Gy [40–110]. One patient presented grade 5 dyspnea and pneumonitis leading to death due to decompensation of emphysema. Two patients presented hemoptysis. The median follow-up was 23.3 months [12.6–31.1]. There was no significant difference in the train cohort between patients with local relapse and without, according to the tumor localization (centrally located in 43% vs 13% and peripherally located in 57% vs 87% respectively, *p* = 0.06) or the type of tumor (lung tumor in 79% vs 70% and lung metastasis in 21% vs 30%, respectively, *p* = 0.71).

### 4.2. Risk Factors for Local Recurrence and Score

**Figure 2:**
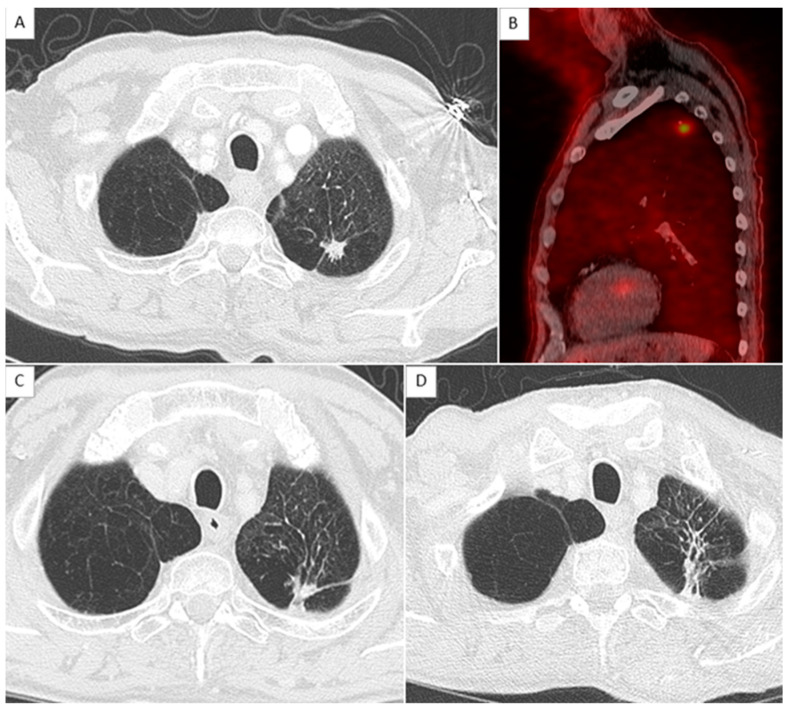
Example of the absence of local relapse with score at 0. (**A**,**C**,**D**) show axial chest CT in parenchymal window with an 11 mm nodule in the left apex corresponding to an adenocarcinoma, before, 1 year, and 2 years after stereotactic radiation therapy, respectively. (**B**) shows a sagittal reconstruction of merge PET and CT with a hypermetabolism of the nodule measured as SUV_max_ = 4.2.

**Figure 3:**
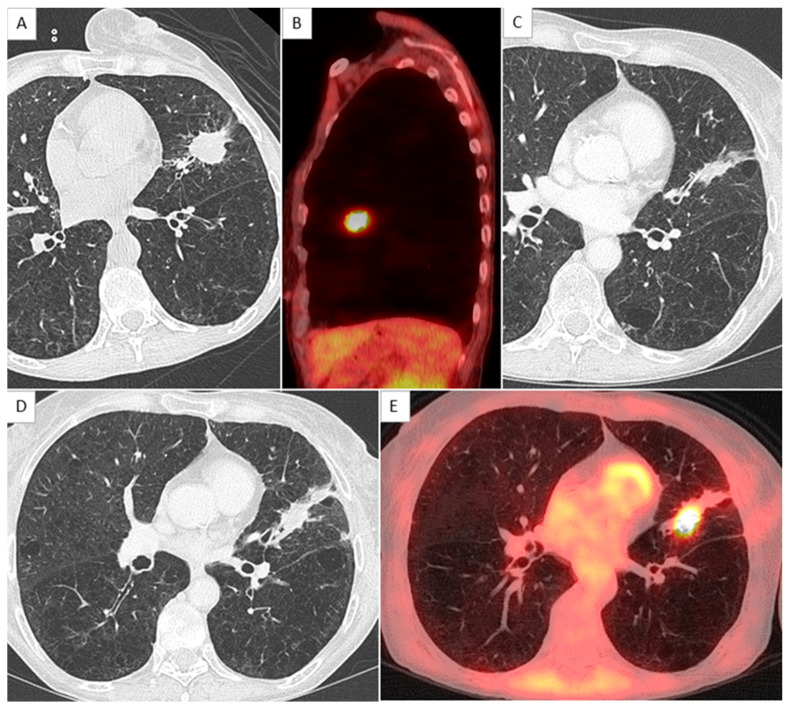
Example of local relapse in a female patient with score at 3. (**A**,**B**) show a lingular mass on axial chest CT and sagittal merge PET and CT, diagnosed after sampling as an adenocarcinoma, with hypermetabolism quantified as SUVmax = 8.0. (**C**) shows the scar 1 year after the treatment, and (**D**,**E**) show a local recurrence 2 years after radiation therapy.

Among the 38 patients, 25 patients were disease-free at the time of inclusion and 13 with local recurrences occurred during the follow-up (Figure 2 and Figure 3). Four of the 13 had a biopsy-proven relapse. BED_10_ was not a significant risk factor for local relapse in the univariate analysis (OR = 0.97 CI 95% (0.94–1.01), *p* = 0.13). Five variables were significantly associated with local recurrence from the univariate analysis and were combined to create an aggregate score ranging from zero to five points. The OR and 95% CI for the five significant risk factors are detailed in Table 2. The area under the ROC curve (AUC) was 0.907 for the whole cohort. The cut-off value, defined by the ROC curve to have the highest values of sensitivity and specificity of the five-variable score according to the Youden index, was 2.5. The sensitivity and specificity for a 2.5-point threshold were 100% and 88%, respectively.

**Figure 4:**
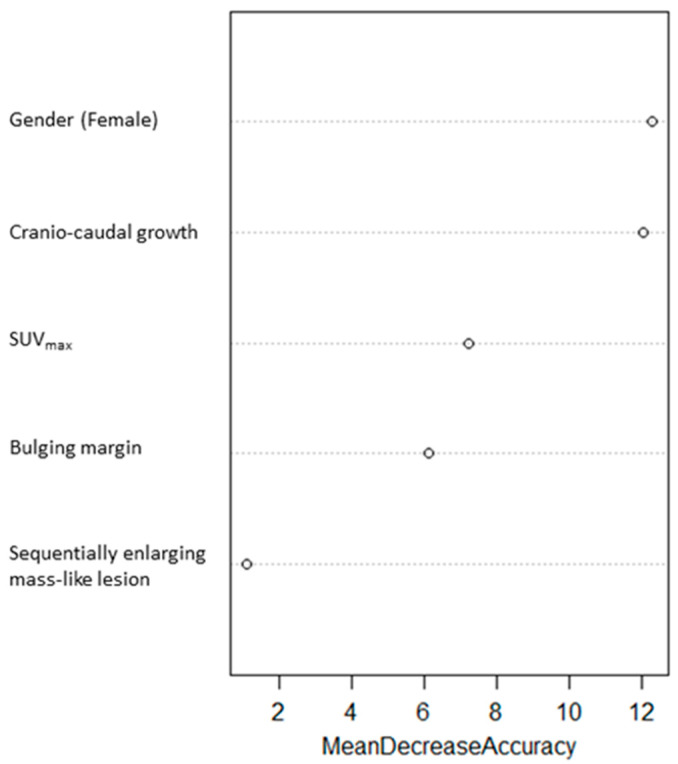
Importance of each variable in the score derived from a Random Forest model.

A Random Forest predictive model of local relapse was trained by the five-item score, and the importance of variables were estimated. Gender (female), craniocaudal growth, SUV_max_, bulging margin, and sequentially enlarging mass-like lesion were ranked from the most to the least important in the model, respectively (Figure 4).

The threshold was then tested on the independent validation cohort of ten patients. The score results for each patient of the validation cohort and the matrix of confusion are presented in Table 3. On this validation cohort, the sensitivity and specificity were 100% and 88%, respectively.

### 4.3. Overall and Local Recurrence-Free Survival

**Figure 5:**
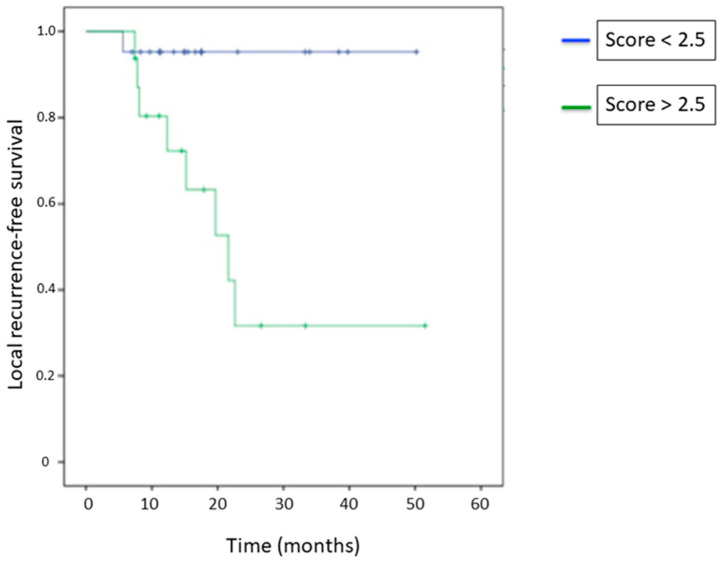
Local recurrence-free survival curves according to the threshold of 2.5.

The local recurrence-free survival was statistically different between the patient group with scores < 2.5 and the patient group with scores > 2.5 (*p* = 0.006) (Figure 5). The mean local recurrence-free survival for those two groups were 48 and 27 months, respectively. A median of local recurrence-free survival was not achieved for patients with a test score < 2.5. For those with a score > 2.5, the median was 22 months.

The overall survival at 1 and 3 years was 88.9% and 42.2%, respectively. The local recurrence-free survival at 1 and 3 years was 89.1% and 62.5%, respectively. A median of local recurrence-free survival was not reached. The median of distant relapse-free survival was 30 months (95% CI) [16–43].

## 5. Discussion

This study on SBRT for lung tumors proposes a new local recurrence predictive score on five points, based on pre- and post-treatment clinical and imaging parameters. From this score, resulting in the aggregation of significant risk factors for local recurrence, the threshold of 2.5 was defined and tested on an independent validation cohort, reaching a sensitivity and specificity of 100% and 88%, respectively.

The assessment of the evolution of local lesions frequently remains difficult due to the diffuse presentation of radiation-induced inflammatory reactions on chest CT. Currently, these radiological alterations could mimic a relapse. Similar controversies are reported for FDG-PET hypermetabolism in the case of radiation-induced lung injury or tumor relapse, which are difficult to differentiate and seem impossible to differentiate within the first 6 months and even up to one year [17]. For these reasons, the diagnosis of cancer relapse is one of the limitations in the different studies reporting follow-up after lung SBRT [18].

Few studies focused on a predictive score of local relapses after SBRT for lung tumors and are more interested in the evaluation of pre-treatment FDG-PET to predict outcomes. These results are in line with studies reporting the predictive value of pre-treatment FDG-PET and showing the predictive role of SUV_max_, with a formerly defined cut-off of 6 to determine high- and low-risk groups, and a 93% and 42% local control rate at 2 years [19]. More recently, tumor heterogeneity from textural radiomic parameters, especially “entropy” extracted from FDG-PET, reached an AUC of 0.872 in predicting local relapse [20]. However, the predictive role of these FDG-PET radiomics features are difficult to use in daily practice, as the algorithms trained in each study are often not shared publicly online and have not been sufficiently validated on external datasets, even if “entropy” and SUV_max_ seem to be strong predictors on pre-treatment FDG-PET [21]. For this reason, we aimed to use parameters easily measured from FDG-PET in daily practice to build the score.

A study reported an approach of CT criteria alone in the context of early lung cancer treated by SBRT, and again found that the enlarging criteria on chest CT scan are the best imaging parameters to predict recurrence [22]. A radiological predictive score of relapses is still painfully lacking. In our study, in addition to pre-treatment FDG-PET parameters, which are known predictive factors, we combined these with radiologic follow-up CT criteria to create a mixed predictive score of relapses. Moreover, in the current study, a training validation protocol was followed to validate the score established from the training cohort. Except in the study reported by Dissaux et al. [23], no study followed this schema. These scores need to be tested on validation datasets, ideally from independent centers.

Magnetic resonance imaging (MRI) criteria could be interesting in follow-up, and recently, a few radiation oncology teams began to evaluate the benefit of thoracic T1 perfusion by dynamic contrast enhancement and diffusion-weighted sequences after lung SBRT [24]. The dual-energy CT or perfusion CT, using high temporal resolution and a large field of view from multiple detectors available with the new generation of CT, will be interesting in the detection of relapse after local treatment [25].

We have reported a sensitivity of 100%, including clinical parameters such as gender. In this study, the female gender was found to be an unfavorable predictive parameter. Although gender is not often reported as an unfavorable clinical parameter, a case series reports three out of four patients who developed a late relapse (more than five years) after SBRT for lung cancer were females [26]. Interestingly, Oikonomou et al. also reported a clinical and radiomic score for relapse prediction after SBRT where being female was an unfavorable criterion [21].

One limitation of this study is the retrospective design and lack of histological proof of relapse for all patients. To mitigate this problem and decrease the level of error, all of the CT and FDG-PET imaging at every time point has been reviewed sequentially by a radiologist physician and nuclear physician, and then evaluated by the local multidisciplinary team. The diagnosis of local recurrence after lung SBRT is very challenging. The gold standard is biopsy, but lung SBRT is oftentimes indicated for patients with a high risk of lung puncture (for instance, extensive emphysema with risk for long drain aspiration or pulmonary hypertension). A second limitation is that the three items on post-therapy CT are related to scar growth and are also the definition of local relapse by the reader. To limit this bias, we used an anonymously reviewed independent validation cohort. The third major limitation is the small sample size due to monocentric collection. It is therefore necessary to continue working on this predictive score with a larger number of patients.

## 6. Conclusions

This score combining FDG-PET and chest CT criteria, created from a retrospective cohort, is a new tool for patient follow-up after lung SBRT. The interest was in the combination of FDG-PET pre-treatment and chest CT post-treatment parameters. Its application on an independent validation cohort reached a sensitivity and specificity of 100% and 88%, respectively. Evaluations of this score on larger external validation cohorts are needed.

## Figures and Tables

**Table 1:** Patient characteristics.

Characteristics	Train Cohort (N = 38)	Validation Cohort (N = 10)	*p*-Value
Gender			
Male	28 (74%)	4 (40%)	0.06
Female	10 (26%)	6 (60%)	
Age (years)	70 ± 12 [49–90]	66 ± 11 [55–80]	0.34
Tobacco use	25 (66%)	10 (100%)	0.04
Diabetes	7 (18%)	1 (10%)	0.99
Cardiovascular diseases	27 (71%)	7 (70%)	0.99
ECOG performance status			
0	17 (45%)	2 (20%)	0.06
1	9 (24%)	5 (50%)	
2	4 (11%)	3 (30%)	
3	0 (0%)	0 (0%)	
4	0 (0%)	0 (0%)	
Unknown	8 (21%)	0 (0%)	
Lung tumors			
NSCLC	27 (71%)	9 (90%)	0.22
Metastases	11 (29%)	1 (10%)	
Tumor location			
Central tumor	11 (29%)	3 (30%)	0.95
Peripheral tumor	27 (71%)	7 (70%)	
Lobe			
Superior lobe	20 (53%)	3 (30%)	0.03
Middle lobe or lingula	8 (21%)	0 (0%)	
Inferior lobe	10 (26%)	7 (70%)	
Histological biopsy proof of malignancy	11 (29%)	8 (80%)	
Adenocarcinoma	6 (16%)	8 (80%)	0.08
Squamous cell carcinoma	4 (11%)	0 (0%)	
Renal adenocarcinoma metastasis	1 (3%)	0 (0%)	
Previous thoracic radiotherapy	4 (11%)	2 (20%)	0.42
Local recurrence	13 (34%)	2 (20%)	0.39
Reason of not surgery treatment			
Surgical contraindication	27 (71%)	10 (100%)	0.15
- Respiratory insufficiency	17 (37%)	6 (60%)	
- Others	10 (26%)	4 (40%)	
Patient refusal	2 (5%)	0 (0%)	
Unknown	9 (24%)	0 (0%)	

Note: Results are expressed as numbers (percentage) or median ± IQR [min–max]. ECOG: Eastern Cooperative Oncology Group, NSCLC: non-small-cell lung cancer.

**Table 2:** Variables significantly associated with local recurrence and risk score calculation.

Variables	Odds Ratio	95% CI	*p*-Value	Risk Score Calculation	
Sequentially enlarging mass-like lesion	12.0	1.2–120.1	0.034	Yes	1
				No	0
Craniocaudal growth	19.2	1.8–199.9	0.013	Yes	1
				No	0
Bulging margin	10.6	1.5–76.1	0.019	Yes	1
				No	0
SUV_max_ ≥ 5.5	11.0	1.6–73.9	0.014	Yes	1
				No	0
Gender (Female)	11.0	1.6–73.9	0.014	Yes	1
				No	0
					**Total: …/5**

**Table 3:** Score results for each patient in the validation cohort and confusion matrix.

Patients	Scores	Defined by the Score: Local Relapse Yes/No		Real Outcomes	
1	0	No		No relapse	
2	1	No		No relapse	
3	3	**Yes**		**Local relapse (biopsy-proven)**	
4	0	No		No relapse	
5	2	No		No relapse	
6	1	No		No relapse	
7	5	**Yes**		No relapse	
8	3	**Yes**		**Local relapse (biopsy-proven)**	
9	0	No		No relapse	
10	1	No		No relapse	
				True relapse	No relapse
			Predicted relapse	2	1
			Predicted no relapse	0	7

## Data Availability

Data are available on request.

## Abbreviations

BED	biologic effective dose
CT	computed tomography
FDG-PET	fluorine-18-fluorodeoxyglucose positron emission tomography
MTV	metabolic tumor volume
NSCLC	non-small-cell lung cancer
ROC	receiver operating characteristics
SBRT	stereotactic body radiation therapy
SUV	standardized uptake value
